# Monitoring of the Red-Belted Clearwing Moth, *Synanthedon myopaeformis*, and its Parasitoid *Liotryphon crassiseta* in Apple Orchards in Yellow Moericke Traps

**DOI:** 10.1673/031.013.0401

**Published:** 2013-01-08

**Authors:** Marek Bąkowski, Hanna Piekarska-Boniecka, Ewa Dolańska-Niedbała

**Affiliations:** 1 Department of Systematic Zoology, Adam Mickiewicz University, Umultowska 89, 61–614 Poznań, Poland; 2 Department of Entomology, University of Life Sciences in Poznań, Dąbrowskiego 159, 60–594 Poznań, Poland

**Keywords:** Ichneumonidae, pest, Sesiidae

## Abstract

This study was conducted in 2008–2010 in three apple orchards in western Poland and involved a massive catch of the red-belted clearwing moth, *Synanthedon myopaeformis* (Borkhausen) (Lepidoptera: Sesiidae), and its parasitoid *Liotryphon crassiseta* (Thomson) (Hymenoptera: Ichneumonidae) in yellow Moericke traps. The flight time for both species was correlated and fell in the first half of July. However, the correlation between the occurrences of both species was statistically significant only in 2008, when most specimens were caught. A total of 7960 *S. myopaeformis* were caught, with a 2:1 male:female sex ratio, and 415 adult *L. crassiseta*. No correlation between the numbers of *S. myopaeformis* and *L. crassiseta* in relation to age, variety of trees, or orchard surface area was noted. Significant differences between the catches of *S. myopaeformis* and *L. crassiseta* were reported in particular years. Furthermore, clear differences in the yields of *S. myopaeformis* and *L. crassiseta* between traps situated in the orchard and those on its edges were recorded, particularly in the orchard surrounded by cultivated fields. Yellow pan-traps could be used more widely in order to monitor and control the abundance of *S. myopaeformis*, especially by catching its females.

## Introduction

In Europe the red-belted clearwing moth, *Synanthedon myopaeformis* (Borkhausen) (Lepidoptera: Sesiidae), was regarded as a secondary pests of apple trees, weakened by other factors until the 1960s. Since that time, it has become a significant pest, which can be attributed to changes in apple production technology (Balazs et al. 1996). It has also been reported to attack apple trees in Germany (Dickler 1976), Italy (Balazs et al. 1996), and Bulgaria (Kutinkova et al. 2006). It has also been reported as the most common sesiid in Jordan and Middle East countries (Abd Elkader and Zaklama 1971; Ateyyat 2005; Ateyyat and Al-Antary 2006).

The larvae of *S. myopaeformis* live for one year between the bark and wood of various fruit trees, especially trees in the subfamily Maloidea C. Weber (Rosaceae), mainly the apple tree, *Malus*, but sometimes also *Pyrus* spp., *Crategus* spp., *Sorbus* spp., *Prunus* spp., *Eriobotrya japonica* Thunb., and several other closely related plant species such as *Hippophae rhamnoides* L. (Laštůvka and Laštůvka 2001). The larvae form a shallow corridor in cankers and in cracked trunks and branches. Pupation occurs in a dense cocoon made of sawdust and silk.

*S. myopaeformis* is difficult to control because its adults have a long emergence period and the larvae develop inside the trunk and thick branches, where they are relatively wellprotected. Failure to prevent injury to *S. myopaeformis* can lead to reduced tree vigor and yield, and infested trees are much more vulnerable to infestations of fungal diseases such as canker (Iren et al. 1984). Control methods include targeting larvae with insecticides of the pyrethroid group (deltarnetrine (Decis 2,5EC) and alphacypermetrin (Fastac 100EC)) or other chemicals such as motor oil (Erler 2010) and pear ester with acetic acid (Tóth et al. 2012). More natural methods based on biological control include bacteria (Shehata et al. 1999), nematodes (Kahounova 1991), and fungi (Cossentine 2010). The results of a study on the effects of three apple rootstocks on the development of *S. myopaeformis* were reported by Ateyyat (2006).

Recently, the application of sexual pheromones in monitoring Sesiidae, particularly & *myopaeformis*, has significantly increased (Voerman 1978; Voerman and Van Deventer 1984; Trematerra 1993; Kutinkova et al. 2006). The method seems very effective at defining the flight dynamic and monitoring the population. However, sex pheromones attract only males and thus the method is not the optimal one to control Sesiidae moths. A valuable indicator of *S. myopaeformis* occurrence is the presence of the empty pupal skins, which remain protruding from the tree.

An essential aspect in biological control of *S. myopaeformis* is the role of parasitoid insects, which reduce *S. myopaeformis* abundance. Studies on entomoparasitoids of *S. myopaeformis* were conducted in Germany by Söntgen and Sengonca (1988) and are among only a few studies of parasitoid entomofauna of Sesiidae. One parasitoid species controlling the number of larval-stage *S. myopaeformis* is ectoparasitoid *Liotryphon* crassiseta (Thomson) (Hymenoptera: Ichneumonidae: Pimplinae), reported from this host by Herting (1973) and Söntgen and Sengonca (1988).

During preliminary testing of using Moericke traps to catch Ichneumonidae in the vicinity of Poznań (western Poland), significant catches of both sexes of *S. myopaeformis* were observed. This finding was surprising because never before had a more numerous catch of this species in the yellow pan-traps been observed. The aim of the study was to define the flight dynamics and spatial distribution of *S. myopaeformis* and its larval ectoparasitoid *L. crassiseta* by using yellow Moericke traps placed both on the edges and inside apple orchards of integrated production that varied in terms of age and area.

## Materials and Methods

### Study area

The research was conducted in 2008–2010 in the following orchards located in the vicinity of Czempiń, Wielkopolska (western Poland):

1. Apple orchard I, Głuchowo (UTM - XT 18; 52.17466° N, 16.71173° E). Forty-hectare area. Research was conducted in 3-hectare plots with 15-year-old apple trees of the following cultivars: Gala, Ligol, Cortland, Paulared, Red Delicious, and Golden Delicious. The orchard was surrounded by cultivated fields of sweet corn in 2008, oats in 2009, and triticale in 2010.

2. Apple orchard II, Gorzyczki I (UTM XT27; 52.10106° N, 16.81199° E). Twentyhectare area. Research was conducted in 5hectare plots with 15-year-old apple trees of the following cultivars: Paulared, Red Delicious, Golden Delicious, and Jonagold. The orchard was surrounded by shrubberies, namely thicket phytocenoses of *EvonymoPrunetum spinosae* and *Querco-Ulmetum* forest, herb communities, and ruderal plant communities.

3. Apple orchard III, Gorzyczki II (UTM - XT27; 52.10208° N, 16.81451° E). Tenhectare area. Research was conducted in 2hectare plots with 20-year-old Golden Delicious apple trees. The orchard bordered a road overgrown with plants typical of the *Rhamno-Prunetea* class.

In all the studied orchards, the trees grew 1.4 m apart from each other in rows 3 m apart. Between the trees, fallow land was maintained and the rows of trees were divided by swards. The orchards followed integrated fruit production policy.

### Methods

During the study, adults were caught in Moericke yellow traps (Moericke 1953). The trap was a yellow, plastic pan filled with water and ethylene glycol as a preservative that also lowers the surface tension. The pans were 18 cm in diameter and 11 cm deep. Twenty pans were placed 1–1.5 m above the ground at each site. Ten traps were located inside the orchard, and 10 were located 5 - 7 m from the orchard's edge. The traps were placed up to 10 m from each other. Specimens were collected over ten-day periods. Insects caught in ten pans during ten days constituted one sample. The traps were placed in the orchards from April 1^st^ to October 31^st^ in each study year. One hundred and sixty-two samples were analyzed in the study of the flight period of *S. myopaeformis* from May 10th to August 20^th^ . For *L. crassiseta*, 372 samples were analyzed from April 1^st^ to October 31^st^. Nonparametric statistics (Kruskal-Wallis test, Mann-Whitney test, Spearman rank correlation) were used because data exihibited an abnormal distribution that a data transformation could not fix. All the statistical analyses were performed using the Statistica software program (Statsoft, http://www.statsoft.com). Average monthly temperatures and precipitation data were obtained from the meteorological station in Turwia (western Poland).

## Results

In the three studied years, a mass occurrence of *S. myopaeformis* was observed in the orchards. A total of 7960 specimens of the species were caught with a 2:1 male:female sex ratio. Also, a proportionally high abundance of its parasitoid, *L. crassiseta*, was recorded, with a total of 415 specimens. All the studied orchards yielded similar abundances *of S. myopaeformis* (Table 1, 2).

No significant statistical differences were recorded between the catches in the three orchards, which varied in terms of area and tree age: H = 3.47; N = 162; *p* = 0.176. However, clear differences in the catches of *S. myopaeformis* were found between particular years: H = 7.85; N = 162;*p* = 0.019. Visible variation in the catches of *S. myopaeformis* was noted between the pans located in the orchard and those outside it, and was proven bv the analysis of the three orchards: U = 2.322; z = 3.21;*p* < 0.01 (Table 2).

**Table 1.**
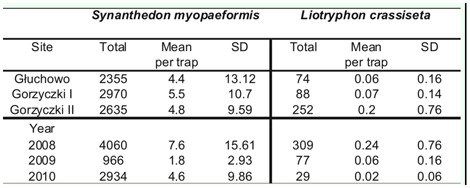
Total and mean number per trap of *Synanthedon myopaeformis* and its parasite *Liotryphon crassiseta* captured in yellow pan-traps in three apple orchards near Czempiń (western Poland) in 2008–2010.

**Table 2.**
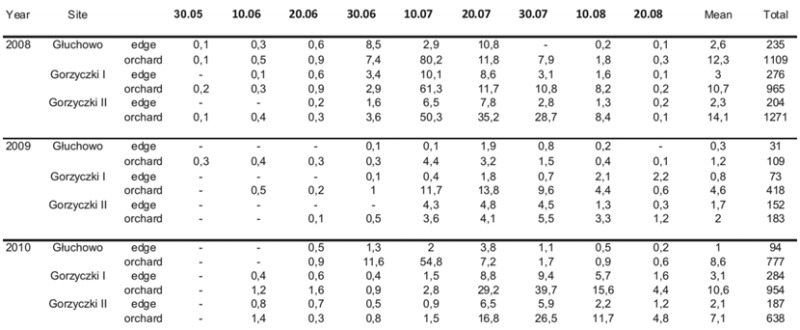
Total and mean number per trap of *Synanthedon myopaeformis* captured in yellow pan-traps located on edges and within three apple orchards near Czempiń (western Poland) in 2008–2010.

The biggest differences were noted in apple orchard I, where the orchard was surrounded by fields. Smaller or no differences occurred when the orchards were surrounded by more varied trees, bushes, and herbs. Statistical significance was found for the catches in apple orchard I, where traps were placed in the orchard and in the field (U = 230.5; z = 2.32; *p* < 0.05 and in apple orchard II, where pans were hung in the orchard and in the shrubberies outside the orchard (U = 248; z= 2.02; *p* < 0.05). In apple orchard III, where pans were located in the orchard and by the road, no significant differences were found (U = 312.5; z = 0.9;*p* > 0.05).

The catches of *L. crassiseta* in the orchards were varied (Table 1, 3). However, as for & *myopaeformis*, no significant statistical differences were found between the catches in particular orchards: H = 3.8; N = 372; *p* > 0.05.

Significant statistical differences in the catches in particular years were found for this species: H = 20.81; N = 372; *p* < 0.01). However, Dunn's post hoc test showed that the differences between the catches in 2009 and 2010 were insignificant.

The catches of *L. crassiseta* in the orchard and on its edges were similar to those of *S. myopaeformis* (Table 2, 3). Significant differences were noted only in apple orchard I, i.e., in the orchard-field combination: U = 11,242; z = 3.62; *p* < 0.01. In the remaining orchards and their vicinity, the differences were insignificant: U = 1813.5; z = 0.83; *p* > 0.05, and U = 1808.5; z = 0.86; *p* > 0.05.

An analysis of correlations between the occurrences of *S. myopaeformis* and *L. crassiseta* in the three studied orchards indicated a significant difference only in 2008 (rs = 0.75; df = 19; *p* < 0.01), while no significant difference was indicated in 2009 (rs = 0.39; df = 19; *p* > 0.05) or 2010 (rs = 0.04; df = 19; *p* > 0.05). The lack of correlation in the last two years might be explained by a low abundance of *L. crassiseta*.

**Figure 1.**
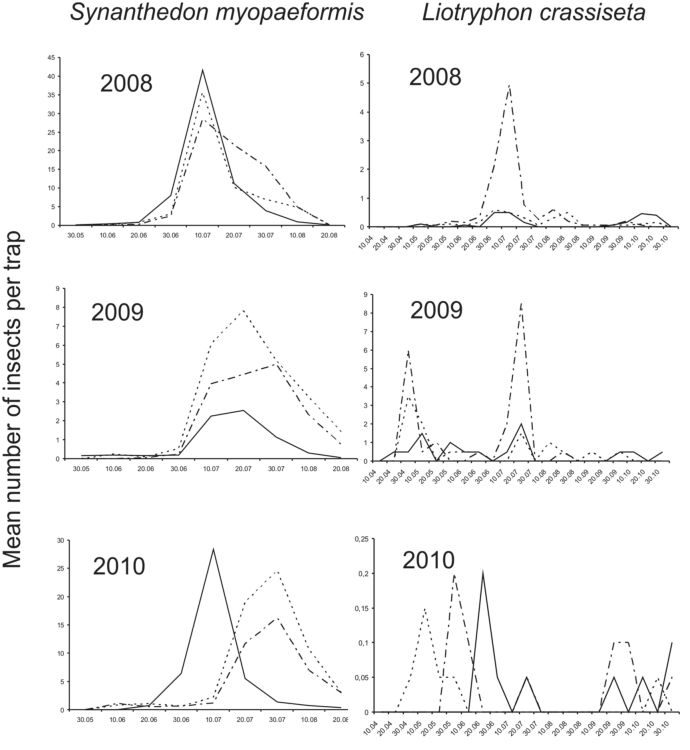
Mean number per trap of *Synanthedon myopaeformis* and *Liotryphon crassiseta* captured in yellow pan-traps in three commercial apple orchards near Czempiń (western Poland) in 2008–2010. High quality figures are available online.

*S. myopaeformis* occurred from May to August, with a peak in the first twenty days of July in all three years of the study (Figure 1). *L. crassiseta* occurred in apple orchards from the first ten-day period in May to the second ten-day period in October, with the maximum catch also occurring in the first twenty days of July (Figure 1).

**Table 3.**
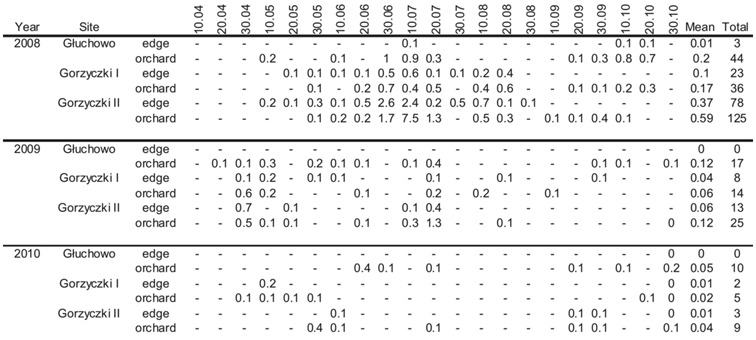
Total and mean number per trap of *Liotryphon crassiseta* captured in yellow pan-traps located on the edges and within three apple orchards near Czempiń (western Poland) in 2008–2010.

## Discussion

Due to difficulties in breeding xylophagous species of Sesiidae and the shortcomings of study-methods, very little information has been collected on the proportion of sexes in the species. Current research has proved that there are an average of 2 *S. myopaeformis* males for 1 female.

The most recent research done in Hungary (Tóth et al. 2012) indicated that lures with pear ester and acetic acid (PEAA) were attractive to *S. myopaeformis* no matter whether the two compounds were provided in separate dispensers or mixed together in a single dispenser, and a large percentage (40– 80%) of *S. myopaeformis* caught were females. However, the results of the present study yielded a better-founded sex ratio than those that used PEAA as an additional attractant.

**Figure 2.**
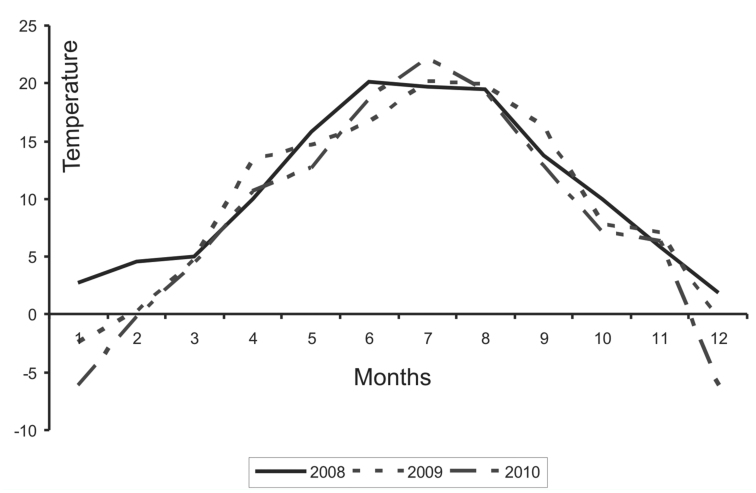
Distribution of mean monthly temperatures in 2008–2010. Data from meteorological station in Turwia (western Poland). High quality figures are available online.

In the present study, the most specimens of *S. myopaeformis* were recorded in 2008, and a significant drop was seen in the following two years. The lower number of specimens in 2009 and 2010 could have resulted from factors such as weather conditions (Figure 2). In January and February 2008, the average mean temperatures were considerably higher than in 2009 and 2010, which undoubtedly could have affected the survival of caterpillars in winter. On the other hand, a considerable decrease in the population size in 2009 and 2010 might have resulted from the large catch in 2008.

One of the more significant results of the study shows the variety in catches of both *S. myopaeformis* and *L. crassiseta* between the pans located in the orchards and those on the orchards’ edges. The biggest differences were noted in apple orchard I, where the orchard was surrounded by fields. Smaller or no differences occurred when the orchards were surrounded by more varied trees, bushes, and herbs. The results obtained indicate that both *S. myopaeformis* and *L. crassiseta* do not tend to expand outside of their larvae or adult feeding area. It is widely known that adult *S. myopaeformis* feed on nectar and can be found on flower plants, which did not grow in the fields around the studied orchards.

Such an abundant catch of the parasitoid, especially *L. crassista*, corroborates the influence of yellow color both on Sesiidae and Ichneumonidae. It also proves similar kinds of behavior for these heliophile insects. The fact that male *S. myopaeformis* prefer pheromonebaited yellow and green traps was confirmed by Tremeterra (1993). Similar relations were found for *Vitacea polistiformes* (Harris). Male *V. polistiformes* prefer green and yellow pheromone-baited traps, but do not appear to distinguish between these two colors, which have a similar spectral reflectance (Roubos an Liburd 2008). This attraction to yellow and green seems to be a permanent feature of Sesiidae species, but studies of female color preferences are necessary.

As was confirmed by the research, the peak occurrence of *S. myopaeformis* happens at the beginning of July. This flight period is also confirmed by the results of a long-standing field study in Poznań (western Poland), in which males were caught in pheromone-baited traps (Bajcowski, unpublished data), as well as earlier results obtained by other researchers (Voerman 1978; Voerman and Van Deventer 1984; Trematerra 1993; Kutinkova et al. 2006). The peak occurrence of *L. crassiseta* was also at the beginning of July (Figure 1). Thus, the occurrence and mass abundance of *S. myopaeformis* and *L. crassiseta* were found to be synchronized, which might suggest that *L. crassiseta* is a significant member of the parasitoid fauna *of S. myopaeformis* and might control its number. The role of the parasite in the control of *S. myopaeformis* was previously indicated in Germany (Söntgen and Sengonca 1988). However, never before has *L. crassiseta* been caught in such large numbers and in correlation with the mass occurrence of & *myopaeformis*. The parasitizing by *L. crassiseta* of another Sesiidae species, *Parathrene tabaniformis* Rott., was reported by Georgiev (2000). Georgiev indicated a high mortality of the pest larvae caused by parasitoids (38.1–55.6%). However, it must be noted that *L. crassiseta* also parasitizes the larvae of other insects, including the moths that develop on apple trees, e.g. *Blastodacna atra* (Haw.) and *Cydia pommonela* L. (Thompson 1957; Aubert 1969).

The present study had the largest recorded catch of Sesiidae in yellow pans. Such an abundant catch of *S. myopaeformis* in yellow traps shows that yellow traps are an effective and inexpensive method of monitoring and controlling the pest abundance. Furthermore, yellow pans catch both males and females, as opposed to pheromone-baited traps, which catch only males. Yellow pans are also an effective method to study the population structure, time, and spatial distribution of *S*. *myopaeformis* and accompanying parasite entomofauna.

This method could be even more effective when combined with additional atractant, e.g., PEAA, and could be a useful female-targeted lure for *S. myopaeformis*. In some cases, a combination of mass trapping and mating disruption would be effective, such as mass trapping of female moths by using kairomones, while using sex pheromones to disrupt male orientation to females (Knight et al. 2002).
